# Adherence to and predictors of EBP in Swiss-German speech-language pathology: the influence of intrinsic and extrinsic factors

**DOI:** 10.3389/frhs.2026.1822320

**Published:** 2026-07-13

**Authors:** Sonja Schäli, Verena Hofmann, Erich Hartmann

**Affiliations:** 1 Institute for Language and Communication, The University of Teacher Education in Special Needs, Zurich, Switzerland; 2 Speech-Language Pathology, Department of Special Education, University of Fribourg, Fribourg, Switzerland

**Keywords:** EBPI, evidence-based practice, implementation science, speech-language pathology, structural equation modelling, Theory of Planned Behavior

## Abstract

**Purpose:**

This study assesses adherence to evidence-based practice (EBP) among speech-language pathologists (SLPs) in German-speaking Switzerland and uses the Theory of Planned Behavior (TPB) to explore the influence of intrinsic and extrinsic predictors on EBP application.

**Method:**

The German version of the Evidence-Based Practice Inventory (EBPI) was used to assess four dimensions of EBP. Additional data on sociodemographic and workplace factors were collected. A total of 2.322 SLPs were invited to complete an online survey and 566 questionnaires were included in the analyses. Path models examined how individual and contextual factors influence EBP application.

**Result:**

Adherence for the four EBP dimensions was moderate to moderate-high, with mean scores ranging from 3.37 to 4.66 on a 6-point scale. Significant differences were observed among the TPB components, F(2.860) = 411.44, *p* < .001, partial *η*^2^ = .421, with attitude scoring higher than subjective norm, perceived behavioral control, and application.

The path model indicated that EBP application was predicted by more positive attitude, stronger subjective norm, and higher perceived behavioral control. Indirect effects of intrinsic and extrinsic factors were found.

**Conclusion:**

The moderate adherence to EBP observed among the surveyed practitioners highlights the need to strengthen intrinsic and extrinsic factors to ensure successful application of EBP in speech-language pathology.

## 1 Introduction

Evidence-based practice (EBP) has become increasingly prominent within the field of speech-language pathology; it first gained prominence in English-speaking countries before spreading to German-speaking and other European countries. This problem-solving concept, which originated in evidence-based medicine (EBM) (1) has been introduced in various healthcare professions and has gained relevance in clinical and school-based speech-language pathology (2–6). Proponents of the use of EBP by speech-language pathologists (SLPs) seek to increase the use of methods that have been demonstrated in research to be effective, useful, and safe. EBP can promote high-quality service delivery and strengthen the profession's credibility and esteem (7, 8).

EBP requires the conscientious, explicit, and judicious integration of a) external evidence, which takes the form of best available research findings, b) internal or clinical evidence, which takes the form of the expertise of the practitioner, and c) patient evidence, which takes the form of the individual preferences, needs, and values of the person with the impairment or health problem (3, 7). In this process, practitioners form the active component. Their task is to acquire, evaluate, and integrate this diverse and equally relevant information in order to make informed decisions that are in the best interest of the client (9).

The use of EBP is challenging and influenced by intrinsic and extrinsic factors. Intrinsic factors refer to person-related characteristics, such as professional experience, academic qualification, and EBP-related knowledge and skills. Extrinsic factors comprise scientific and contextual conditions. Scientific conditions concern the availability, quality, and accessibility of current research evidence, whereas contextual conditions refer to organizational and cultural characteristics of the workplace, such as professional setting, workplace support, and time available for scientific reading. Depending on their specific manifestation, these factors can act as facilitators of or barriers to EBP (10–12).

### 1.1 Intrinsic factors

The application of EBP in everyday practice requires professional expertise and experience, the ability to reflect and make critical judgements, and the EBP-specific knowledge and skills needed to acquire, appraise, apply, and assess evidence. Such competencies must be built as part of training and continuing education and regularly applied and strengthened in practice, otherwise they may be forgotten or used improperly (12–14). From a psychological perspective, a positive SLP attitude towards EBP is important, as attitude can influence behavior. A belief in the beneficial use of evidence for practice, and thus for the client, results in a positive attitude towards EBP. The practitioner's assessment of social pressure within the workplace to demonstrate or avoid displaying EBP-related behavior also plays a role in the use of EBP (15). Finally, the academic qualification and degree of the professional are further relevant factors (e.g., bachelor's, master's or doctoral degree) (10, 12, 13).

### 1.2 Extrinsic scientific factors

Although research in speech-language pathology has progressed in recent decades, further scientific work is needed to provide a high-quality empirical base for the effectiveness and usefulness of various interventions for children and adults. The availability of guidelines, systematic reviews, and original studies on specific questions makes it easier for SLPs to select and use validated methods. It is important that SLPs have unrestricted access to scientific journals and other sources of external evidence at their workplace (3, 7, 9).

### 1.3 Extrinsic contextual factors

Several organizational and work-culture characteristics have a considerable impact on the ways in which EBP is applied. Factors include sufficient personal resources, the provision of further training on the topic of EBP, opportunities and time for informal information sharing and discussions with colleagues and clients, positive attitudes towards EBP by colleagues and superiors and support within the organization, and targeted implementation strategies to promote EBP (8, 11, 12, 16). The necessary infrastructure (computer, internet) and unrestricted access to external evidence (journals, databases) must also be available. Above all, SLPs need sufficient time to search for, evaluate, and integrate external evidence and apply the results in practice, which is essential for the competent and consistent use of EBP, regardless of the professional setting (7, 17).

Despite the significance and efficacy of EBP, its implementation in speech-language pathology has lagged other health professions and continues to face persistent challenges (10, 11). Surveys conducted in the USA, Australia, South Africa, Japan, Malaysia, Saudi Arabia, and Ireland have examined issues related to EBP and have provided valuable insights into its uptake in clinical practice (13, 18–22). Converging evidence suggests SLPs hold a predominantly positive attitude towards EBP (12, 13, 20, 23). However, positive attitudes alone are not sufficient for consistent application in practice. At the individual level, barriers to EBP implementation include an incomplete understanding of the EBP concept in general and insufficient knowledge and skills related to its application (21, 24, 25). The findings indicate a positive correlation between the relevant competencies, such as searching, reading and evaluating scientific studies, and academic qualifications. The correlation is particularly pronounced in relation to EBP-related training and exposure to EBP received during professional education and continuing professional development. Practitioners with more extensive training tend to report greater engagement in EBP and fewer perceived barriers (13, 18, 19, 24).

At the organizational level, the most frequently reported obstacle is a lack of time for EBP-related activities, alongside limited access to high-quality empirical evidence, weak leadership, and an organisational culture that does not sufficiently support EBP (12, 20, 26–28).

The successful implementation of EBP in clinical settings is dependent on a comprehensive understanding of the facilitators and barriers specific to the country or region in which it is being implemented (10). While research into EBP among speech-language pathologists has been subject to extensive investigation in English-speaking countries, including large-scale surveys and theory-informed studies, there is a notable absence of comparable research in German-speaking contexts. To date, the empirical evidence on the utilization of EBP in the domain of speech-language pathology in German-speaking countries is limited. The extant literature consists of two studies with small samples: the German Online Speech Therapy Journal Club (29) and an implementation project conducted at the University Hospital of Basel that included therapists from several professional disciplines (30). The findings of these studies highlighted the significance of training opportunities and structured formats for reflection, such as journal clubs, and reported a generally high level of acceptance of EBP among practitioners. Nevertheless, their descriptive nature, limited sample sizes, and focus on specific local initiatives restrict their generalizability and do not allow conclusions about determinants of EBP behavior at a broader level.

Existing international research on EBP in speech-language pathology and related therapy professions has consistently documented a positive attitude towards EBP alongside substantial challenges in its implementation. A range of surveys have identified recurring barriers, including lack of time, limited access to research literature, insufficient methodological confidence, and organizational constraints. However, the majority of existing studies rely on descriptive analyses or isolated regression approaches and rarely test explicit theoretical models. Consequently, the interrelations between individual attitudes, perceived social norms, perceived behavioral control, and behavioral intentions have remained largely unexplored.

Considering these research gaps, the present study aims to assess adherence to EBP among speech-language pathologists from German-speaking Switzerland and to explore predictors of EBP using a theory-driven, model-based approach grounded in the Theory of Planned Behavior. Building on the findings of earlier research, this study applies structural equation modelling (SEM) to simultaneously examine the relationships between latent TPB constructs and EBP adherence. Specifically, attitude, subjective norm, and perceived behavioral control are examined as direct predictors of EBP application, defined in this study as practitioners’ intention and behavior regarding the use of EBP. Intrinsic and extrinsic factors are examined as potential indirect predictors through their effects on these TPB constructs. The term ‘adherence’ is used to describe the extent to which a person's behavior conforms to professional recommendations (10, 31).

Ajzen's (32) Theory of Planned Behavior (TPB) provides an explanation of human behavior that emphasizes behavioral intention as the central variable. This intention is influenced by three main factors: attitudes towards the behavior, that is to say, a personal evaluation of the behavior as positive or negative; subjective norm, which describes the perceived social pressure to perform or refrain from the behavior; and perceived behavioral control, namely the assessment of one's own ability to perform the behavior in light of possible obstacles or facilitators. These three factors are based on specific beliefs – behavioral, normative, and control beliefs – and directly influence the intention to perform the behavior. According to the TPB, behavioral intention and perceived behavioral control are strong predictors of actual behavior. As Guo and colleagues described, the TPB is not intended to be an exclusive model for predicting intention or behavior; consequently, it is recommended that studies grounded in the TPB maintain a flexible framework to accommodate additional predictors that can effectively capture a substantial proportion of the variance in behavioral intention (15). It has been demonstrated by studies that the TPB is suitable for implementation within the context of evidence-based practice (33–35).

## 2 Research questions

In this context, the present study undertakes the first systematic investigation of evidence-based practice among speech-language pathologists in the German-speaking part of Switzerland. Using the Theory of Planned Behavior as an analytical framework, the study examines adherence to Evidence-Based Practice as well as direct and indirect predictors of its use in clinical practice.

The research questions are:

1. How does self-reported adherence to various EBP dimensions - attitude, subjective norm, perceived behavioral control, intention, and behavior - manifest and differ in the sample of practitioners surveyed?
2. What predictive relationships exist between the dimensions attitude, subjective norm, perceived behavioral control, and the application of EBP principles (i.e., the EBPI dimension “intention and behavior”)?
3. How and to what extent do additional intrinsic and extrinsic factors (i.e., degree, EBP training, workplace, access to evidence) predict the application of EBP via the TPB components?

## 3 Materials and methods

### 3.1 Design and ethical issues

This study employed a cross-sectional e-survey design to examine the role of EBP in clinical decision-making among SLPs. A cross-sectional approach was chosen to capture the current state of EBP implementation at a specific point in time. The Evidence-Based Practice Inventory (EBPI) (36), a psychometrically validated questionnaire, was selected based on predefined criteria, including psychometric properties and applicability to allied health professions. The intended sample was SLPs practicing in a variety of settings in German-speaking Switzerland, including clinics, rehabilitation centers, mainstream and special schools, early intervention services, private practices, and other settings. Individuals in managerial roles who did not also work with patients/clients were excluded from the study because the focus was on the role of the EBP in therapeutic decision-making.

No formal ethical approval was required for the survey of SLPs because it did not involve sensitive personal data, medical interventions, or vulnerable populations. However, the study followed the principles of the Declaration of Helsinki and was conducted in an ethical and responsible manner. Participation in the survey was voluntary and informed consent was obtained prior to data collection. At the start of the survey, participants were informed that all data collected would be stored securely and used solely for scientific purposes in anonymized form. It was also stated that no data would be collected through which any individual could be identified, including workplace name or IP address. Participants were required to click ‘Continue’ to proceed with the survey, thereby consenting to the collection and storage of their data in accordance with the stated privacy policy. Data security measures for the survey included encrypted storage and restricted access.

### 3.2 Instrument

The Evidence-Based Practice Inventory (EBPI) questionnaire, developed by Kaper et al. (31) was employed. This 26-item instrument provides a sound inventory for comprehensively assessing adherence to EBP and identifying barriers and facilitators to EBP. The EBPI was translated and culturally adapted to German by Braun et al. (36), who evaluated both the construct validity and internal consistency of the English and German versions with healthcare professionals. EBPI comprises five dimensions: attitude (a clinician's individual evaluation of EBP), subjective norm (a clinician's estimate of the social pressure to perform or not to perform EBP), perceived behavioral control (the extent to which a clinician feels able to enact EBP), decision making (the extent to which new information reshapes the clinician's current understanding and behavior), and intention and behavior (application: the clinician's aim and actual response, respectively, to applying EBP) (10, 31). In the original TPB framework, intention and behavior are theoretically distinct constructs. However, because the validated EBPI combines them into a single dimension, the present study follows this operationalization and uses the EBPI dimension “intention and behavior” as an indicator of EBP application. Items are presented on Likert scales, ranging from 1 to 6, where the value of 1 is assigned to the most negative option, whilst the value of 6 is assigned to the most positive option.

### 3.3 Recruitment and data collection

The sample was recruited with the support of the Swiss German Speech and Language Therapists professional association (DLV) and data collection took place between December 5 and December 25, 2022. The initial survey invitation was accompanied by an informational letter, followed by a reminder email that was sent two weeks later. The survey was conducted using LimeSurvey, an online survey platform, and the survey link was distributed via email by the DLV to 2.322 members.

To facilitate accessibility and maximize participation, a general open-access survey link was used instead of individualized access keys. Because the survey was accessible via a public link, there was a potential risk of multiple submissions by the same individual. To mitigate this, LimeSurvey's built-in feature “set a cookie to prevent repeated participation” was enabled. This measure prevented respondents from completing the survey multiple times using the same browser and device. However, it was technically possible to submit multiple responses from different devices (e.g., personal and work computer).

### 3.4 Data analysis

#### 3.4.1 Adherence to EBP

Based on participants’ ratings of the respective items, scale scores were calculated and descriptively analyzed (*M*, *SD*, range) for the four EBPI dimensions attitude, subjective norm, perceived behavioral control, and intention and behavior (Research Question 1). In addition, Cronbach's alpha was determined for each dimension. For psychometric and theoretical-conceptual reasons (insufficient internal consistency, lack of relevance to the TPB), the EBPI dimension *decision-making* was not included in subsequent analyses. To interpret self-reported EBP adherence level, we used Braun et al.'s proposed categories (10): 1-2 = *low adherence*, 3-4 = *moderate adherence*, 5-6 = *high adherence*. In order to statistically analyze differences between the various EBPI dimensions (dependent samples), a repeated measures ANOVA was performed. If the Mauchly test indicated a violation of the condition of sphericity, then the degrees of freedom were corrected. Pairwise *post-hoc* comparisons with Bonferroni adjustment were used to test the significance of- differences between the means of the different EBPI dimensions. An alpha error level of 5% was set for all statistical analyses (two-tailed).

#### 3.4.2 Predictors of EBP

Regarding Research Question 2, analyses examined whether EBP application could be predicted by attitude, subjective norm, and perceived behavioral control (according to the TPB). Within the framework of path analysis using the software Mplus (Version 8.6) (37) the direct effects of the three predictors were estimated.

To answer Research Question 3, additional predictors were included in the path analysis to account for workplace context (extrinsic factors) and individual competencies/experience (intrinsic factors). Within the work environment, variables included setting (clinic, regular school, special school, other settings), access to external evidence, and time for scientific reading. Individual-level variables included professional experience, exposure to EBP in training or further education, and highest professional degree (diploma/bachelor vs. master/PhD). Analyses examined possible direct effects of these variables on behavior/intention and possible indirect effects via attitude, subjective norm, and perceived behavioral control.

## 4 Results

### 4.1 Participant characteristics

A total of 566 questionnaires were included in the analysis, for an overall response rate of 24.38% based on the number of invitations sent (*N* = 2.322) or 50.45% based on the number of accesses to the survey homepage (*N* = 952). Table 1 summarizes the demographic and professional information of the respondents. The mean age was 42.56 years (*SD* = 11.84), and the vast majority was female (96.82%). These numbers are consistent with information from the professional association DLV and universities with speech-language pathology programs. Slightly more than half of the respondents worked in preschool and school settings, with 41.9% in regular schools and 14% in special schools. The remaining work environments included clinical facilities for adults (8.7%) and other locations (35.5%, e.g., private practice, early intervention centers).

**Table 1:** Characteristics of the study participants (*N* = 566).

Characteristics	Descriptive statistics
Age; yrs (*M*; *SD*)	42.56 (11.84)
Sex (*n*; *%)*	
- Female	548 (96.82)
- Male	18 (3.18)
Place of employment (*n*; *%)*	
- Regular school	237 (41.9)
- Special school	79 (14)
- Clinic	49 (8.7)
- Other settings	201 (35.5)
Highest professional degree obtained (*n*; %)	
- Diploma/Bachelor's	485 (85.7)
- Master's or PhD	81 (14.3)
Professional experience; yrs (*M*; *SD*)	14.99 (10.04)
EBP in training and/or further education; yes (*n*; *%)*	377 (66.61)
Access to external evidence (*n*; *%)*	360 (63.60)
Time (min./week) for scientific reading; min./w. (*M*; *SD*)	23.19 (38.4)

In German-speaking Switzerland, the primary qualification in Speech-Language Pathology is a bachelor's degree, which is sufficient to work in healthcare and educational settings. Further academic qualifications are therefore less commonly pursued. This trend is reflected in the data: 85.7% of respondents held a bachelor's degree, whereas only 14.3% had attained a master's degree or Ph.D. Participants’ mean professional experience was 14.99 years (*SD* = 10.04). Two-thirds of respondents reported having received training and/or further education on EBP. Additionally, 63.60% reported access to external evidence such as research studies or guidelines. On average, participants dedicated 23.19 minutes per week (*SD* = 38.4) to scientific reading, indicating a high degree of variability in engaging with academic resources.

### 4.2 Adherence to EBP

Table 2 presents the descriptive results for the four EBPI dimensions. The data indicate that the scale mean values corresponded to moderate (subjective norm, perceived behavioral control, intention, and behavior) or moderate to high adherence (attitude) in accordance with the criteria outlined in Braun et al. (10). There were considerable inter-individual differences in the ratings for all dimensions, as documented by the scale ranges. The Cronbach's alpha values for attitude, subjective norm, and perceived behavioral control were comparable to those found by Braun et al. (36) (*α* = 0.83 – 0.90); internal consistency for intention and behavior was lower (*α* = 0.76 compared to *α* = 0.83) but still acceptable.

**Table 2:** Descriptive statistics for the four EBPI dimensions (*N* = 566).

EBPI dimensions	Number of items	M (SD)	Min.	Max.	Cronbach's *α*
Attitude	8	4.66 (.81)	1.50	6.0	.90
Subjective norm	5	3.89 (.95)	1.00	6.0	.83
Perceived behavioral control	6	4.15 (1.00)	1.00	6.0	.90
Intention and behavior	4	3.37 (.90)	1.25	6.0	.76

When analyzing differences between the four EBPI dimensions, we first checked whether the assumption of sphericity would be violated in a repeated-measures ANOVA and found that it was (Mauchly-*W* [5] = .909, *p* < .001). Therefore, the degrees of freedom were corrected using Huynh-Feldt estimates of sphericity (*ε* = 0.953). A significant global effect on the dependent variable was found (*F*(2.860) = 411.44, *p* < .001, partial *η*^2^ = 0.421). Bonferroni-corrected pairwise comparisons indicated significant differences between all dependent variables: The scale mean for attitude (*M* = 4.66; *SD* = 0.81) was higher than for subjective norm (*M* = 3.89; *SD* = 0.95; *p* < .001), perceived behavioral control (*M* = 4.15; *SD* = 1.00; *p* < .001), and intention and behavior (*M* = 3.37; *SD* = 0.90; *p* < .001). Adherence was lower for subjective norm than for perceived behavioral control (*p* < .001) but higher than for intention and behavior, for which a lower adherence was found compared to perceived behavioral control (*p* < .001).

### 4.3 Predictor analysis

**Figure 1:**
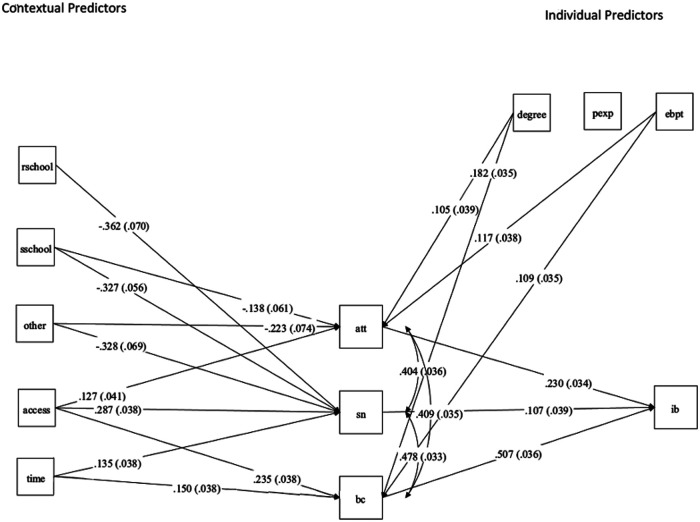
Results of path analysis to predict EBP intention and behavior (arrows indicate significant effects). att, attitude; sn, subjective norm; bc, perceived behavioral control; ib, intention and behavior; rschool, regular school (compared to clinic); sschool, special school (compared to clinic); other, other workplace (compared to clinic); access, access to external evidence (compared to no access); time, time (min./week) for scientific reading; degree, highest degree obtained (master/PhD compared to diploma/bachelor); pexp, professional experience (years); ebpt, EBP in training/further education (compared to no training).

To answer Research Question 2, we first examined whether attitude, subjective norm, and perceived behavioral control are predictors of EBP application. The path model shown in Figure 1 illustrates all significant effects. Of note, the TPB's three components are all significantly associated with intention/behavior (application) at *p* < .01. The standardized coefficients (0.404–0.478) indicate medium-sized effects.

To answer Research Question 3, individual and contextual predictors that could directly and/or indirectly affect intention and behavior were included in the path model. Figure 1 indicates no significant direct effects of any predictor on the dependent variable. However, several significant indirect effects were found, via the components of the TPB (see Table 3). Regarding contextual predictors, lower attitude and subjective norm toward EBP were found in the work settings “mainstream school”, “special school”, and “other” compared to “clinic”. These effects were indirectly linked to lower use of EBP in these settings. Hence, alongside the influence on attitudes, a stronger norm for practicing EBP was perceived in clinic settings, which in turn predicted intention/behavior. However, perceived behavioral control did not differ between settings. Access to external evidence at the workplace was a positive predictor for all three components (i.e., attitude, norm, and perceived behavioral control), and indirectly related to EBP application. Time available for scientific reading only influenced EBP application through subjective norm.

**Table 3:** Indirect effects of the predictor variables on intention and behavior via attitude, subjective norm, and perceived behavioral control.

		Predictor – Attitude – Intention/Behavior			Predictor – Social Norm – Intention/Behavior			Predictor – Perceived Behavioral Control – Intention/Behavior		
		*B (SE)*	*B/SE*	*p*	*B (SE)*	*B/SE*	*p*	*B (SE)*	*B/SE*	*p*
Contextual predictors
	Regular school^a^	−0.196 (0.129)	−1.517	.129	**−0.278 (0.115)**	**−2**.**428**	.**015**	−0.443 (0.264)	−1.680	.093
	Special school^a^	**−0.326** (**0.152)**	**−2**.**150**	.**032**	**−0.357 (0.144)**	**−2**.**486**	.**032**	−0.395 (0.300)	−1.317	.188
	Other^a^	**−0.380** (**0.139)**	**−2**.**724**	.**006**	**−0.260 (0.109)**	**−2**.**383**	.**017**	−0.324 (0.265)	−1.222	.222
	Access	**0.215** (**0.078)**	**2**.**761**	.**006**	**0.226 (0.088)**	**2**.**587**	.**010**	**0.878** (**0.162)**	**5**.**436**	**<**.**001**
	Time	0.001 (0.001)	0.755	.450	**0.001 (0.001)**	**2**.**168**	.**030**	0.004 (0.003)	1.463	.143
Individual predictors
	Degree	**0.244** (**0.097)**	**2**.**518**	.**012**	–	–	–	**0.933** (**0.192)**	**4**.**860**	**<**.**001**
	Experience	−0.004 (0.003)	−1.234	.217	–	–	–	0.010 (0.006)	1.679	.093
	EBP Training	**0.202** (**0.073)**	**2**.**776**	.**006**	–	–	–	**0.417** (**0.136)**	**3**.**067**	.**002**

Direct and indirect effects were not calculated for individual predictors on/via social norms, since the perception of norms is assumed to depend on contextual factors and not on individual factors.

Significant effects are shown in bold.

a Reference category = clinic.

In terms of individual predictors, a master's or doctoral degree (compared to a diploma or bachelor's degree) was predictive of more positive attitudes toward EBP and stronger perceived behavioral control, which in turn were associated with an increased tendency to use EBP. The same trend was observed for individuals who had completed EBP training in their primary or continuing education compared to individuals who had not completed EBP training. In contrast, professional experience appeared to have no influence on attitude or perceived behavioral control.

## 5 Discussion

This study sought to a) measure EBP adherence among SLPs in German-speaking Switzerland and b) explore the influence of intrinsic and extrinsic factors on EBP implementation. The analyses of survey data from 566 practitioners provide first comprehensive insights into the current status of EBP in Swiss speech-language pathology.

### 5.1 Adherence to EBP

Regarding Research Question 1, the results show that EBP adherence as measured by the EBPI is moderate or moderate to high, with significant differences between the four dimensions. The dimension with the highest mean indicates that the SLPs have a positive attitude towards EBP overall, with considerable inter-individual variance. These results are consistent with previous findings from international studies on SLPs’ attitudes towards EBP (12, 13, 20, 28). For the other three dimensions, adherence was comparatively lower but still moderate. The dimension “perceived behavioral control” showed a higher level of adherence than the dimensions “subjective norm” and “intention and behavior”, with the latter exhibiting the lowest level of adherence. As with attitude, substantial inter-individual variance was observed for these three dimensions.

These results point in the same direction as previous research: Although most SLPs or other professionals have a (rather) positive attitude towards EBP and recognize its benefits for practice, there appear to be intrinsic and extrinsic factors that limit the concrete use of EBP in practice (10, 12, 21, 22, 24).

The moderate EBP adherence observed among Swiss SLPs calls for targeted efforts to improve EBP implementation. The results on factors influencing EBP can help identify starting points for improvement.

Most of the results of the predictor analyses were consistent with theory and the state of research. First, more positive attitude, more positive subjective norm, and greater perceived behavioral control were related to greater EBP application. This finding shows that the TPB is a reasonable theoretical model to apply to the application of EBP by speech therapists (for a detailed discussion of the relationships between the EBP components, see (15). Second, indirect effects of individual and contextual predictors via attitude, subjective norm, and perceived behavioral control were observed.

Regarding contextual predictors, a more positive attitude and subjective norm toward EBP were found for therapists in clinical settings compared to other workplaces (i.e., regular schools, special schools, and other settings), which was indirectly linked to greater EBP application. This result could be related to EBP's origins as a medical concept (1); thus, EBP is likely more established in clinical settings than in other work settings. Other important workplace-related factors were access to external evidence and time available for scientific reading. Previous studies have shown that those factors represent frequent barriers for EBP, as they are often insufficiently available or accessible at the workplace (12). However, if available, these factors facilitate the application of EBP. In terms of individual predictors, a master's degree or PhD (compared to a diploma or bachelor's degree) was predictive of more positive attitudes and greater perceived behavioral control, which in turn were associated with an increase in EBP application. The same was observed for individuals who had completed EBP training in their primary or continuing education compared to those who had not completed such training. In contrast, professional experience was not predictive of EBP application. This finding indicates that theoretical knowledge and practical experience with EBP are crucial for the application of the concept, even more so than professional experience itself. However, our results also imply that in the absence of these favorable factors, the application of EBP is limited. As can be seen in Table 1, for example, most participants did not have a master's degree or a doctorate, which also applies to the general population of speech therapists in Switzerland. Furthermore, less than two-thirds of participants reported access to external evidence and the average time spent reading scientific literature per week was 23.19 minutes. Despite the absence of a definitive criterion for comparison, this period appears to be relatively brief for the review and consideration of scientific evidence, particularly in light of the estimated time required for the posing and answering of clinical questions, which is estimated to range from three to seven hours (38). Thus, the barriers we observed in the Swiss context appear to be similar to those found in international studies.

### 5.2 Limitations and further research directions

This study had several limitations. First, although the sample size is sizable compared to previous surveys on EBP in speech-language pathology, the low response rate of 24.38% (based on the number of invitations sent, *N* = 2.322) means the sample cannot be considered representative. One source of possible sample bias is the underrepresentation of SLPs from clinical settings (8.70%), which does not reflect the proportion of SLPs who work in clinical settings (25%–30%) according to information from the professional association DLV (personal communication, DLV, 2025). Second, the use of a survey to assess adherence to EBP could have created possible distortions in response behavior (e.g., recall bias, social desirability). For example, respondents might have over- or underestimated or inaccurately recalled their attitudes, competencies, and behaviors towards EBP. Moreover, perceived behavioral control was assessed through self-report and therefore reflects practitioners’ subjective confidence and perceived ability to apply EBP rather than objectively measured EBP competence. The study did not include direct measures of research literacy, statistical understanding or critical appraisal skills. Similarly, time available for scientific reading was assessed only in minutes per week and therefore captures the duration, but not the quality, depth, or type of engagement with scientific evidence, nor whether the evidence was critically appraised or integrated into clinical decision-making. The survey was only accessible online, which may have disproportionately increased the participation rate of SLPs with an affinity for digital media and content. Furthermore, participants were self-selected, which may have biased participation towards SLPs with higher levels of interest, positive attitudes, and expertise related to EBP. For all these reasons, a generalization of the results to the entire population of German-speaking Swiss SLPs should be made with caution. Third, the EBPI instrument used in this study focusses mainly on external evidence, whereas EBP is a multidimensional concept that requires the integration of other types of information - clinical expertise, client preferences and values - for decision-making. Finally, due to the cross-sectional, non-experimental study design, causality cannot be established.

Since SLPs’ responses reflect their subjective judgements and interpretations based on practical experience, future studies might benefit from including workplace observations and stakeholder interviews to triangulate survey findings with other information sources (12). Adherence to EBP among SLPs from Switzerland could be measured longitudinally to explore the impact of changes towards better implementation. Intervention studies would also be desirable to determine the effects of measures to promote relevant prerequisites and facilitators for a consistent and effective use of EBP in practice.

## 6 Conclusion

This study provides important first insights into adherence to EBP and its connection to the TPB framework components in speech-language pathology in Switzerland. Although attitudes towards EBP were positive, several person- and workplace-related factors were identified as potential barriers to implementation. EBP application appears to be enhanced by access to external evidence and time available for scientific reading. Furthermore, highest degree attained and previous EBP training appeared to have a positive influence on EBP application. These findings emphasize the importance of strengthening individual- and workplace-related aspects to facilitate EBP uptake in speech-language pathology. Future studies could employ a qualitative or longitudinal approach.

## Data Availability

The raw data supporting the conclusions of this article will be made available by the authors, without undue reservation.
